# Integrated Analysis of Metabolome and Transcriptome Reveals Insights for Low Phosphorus Tolerance in Wheat Seedling

**DOI:** 10.3390/ijms241914840

**Published:** 2023-10-02

**Authors:** Pengcheng Li, Xiaole Ma, Juncheng Wang, Lirong Yao, Baochun Li, Yaxiong Meng, Erjing Si, Ke Yang, Xunwu Shang, Xueyong Zhang, Huajun Wang

**Affiliations:** 1 State Key Lab of Aridland Crop Science / Gansu Key Lab of Crop Improvement and Germplasm Enhancement, Lanzhou, 730070, China; lipengcheng@st.gsau.edu.cn (P.L.); maxl@gsau.edu.cn (X.M.);; 2 Department of Crop Genetics and Breeding, College of Agronomy, Gansu Agricultural University, Lanzhou 730070, China; 3 Department of Botany, College of Life Sciences and Technology, Gansu Agricultural University, Lanzhou 730070, China

**Keywords:** wheat (*Triticum aestivum* L.), low phosphorus stress, metabolome, transcriptome

## Abstract

Low phosphorus (LP) stress leads to a significant reduction in wheat yield, primarily in the reduction of biomass, the number of tillers and spike grains, the delay in heading and flowering, and the inhibition of starch synthesis and grouting. However, the differences in regulatory pathway responses to low phosphorus stress among different wheat genotypes are still largely unknown. In this study, metabolome and transcriptome analyses of G28 (LP-tolerant) and L143 (LP-sensitive) wheat varieties after 72 h of normal phosphorus (CK) and LP stress were performed. A total of 181 and 163 differentially accumulated metabolites (DAMs) were detected for G28CK vs. G28LP and L143CK vs. L143LP, respectively. Notably, the expression of pilocarpine (C07474) in G28CK vs. G28LP was significantly downregulated 4.77-fold, while the expression of neochlorogenic acid (C17147) in L143CK vs. L143LP was significantly upregulated 2.34-fold. A total of 4023 differentially expressed genes (DEGs) were acquired between G28 and L143, of which 1120 DEGs were considered as the core DEGs of LP tolerance of wheat after LP treatment. The integration of metabolomics and transcriptomic data further revealed that the LP tolerance of wheat was closely related to 15 metabolites and 18 key genes in the sugar and amino acid metabolism pathway. The oxidative phosphorylation pathway was enriched to four ATPases, two cytochrome c reductase genes, and fumaric acid under LP treatment. Moreover, *PHT1;1*, TFs (*ARFA*, *WRKY40, MYB4, MYB85*), and *IAA20* genes were related to the Pi starvation stress of wheat roots. Therefore, the differences in LP tolerance of different wheat varieties were related to energy metabolism, amino acid metabolism, phytohormones, and PHT proteins, and precisely regulated by the levels of various molecular pathways to adapt to Pi starvation stress. Taken together, this study may help to reveal the complex regulatory process of wheat adaptation to Pi starvation and provide new genetic clues for further study on improving plant Pi utilization efficiency.

## 1. Introduction

Wheat (*Triticum aestivum* L.) is a worldwide cereal staple crop. The increasing global population requires a growing demand for food, making the continued stability and growth of wheat production critically important [1]. Phosphate (Pi) deficiency not only dramatically reduces the biomass accumulation of wheat, delays heading and flowering, and reduces the number of tillers and spike grains but also inhibits starch synthesis and filling, leading to a significant reduction in yield [2]. Phosphorus (P) is one of the essential elements for plant growth and development. In addition to being a crucial part of cell structural makeup, it also regulates the metabolism and signaling of the plant [3,4]. The deficiency of available Pi in soil is usually mitigated by the application of additional phosphorus fertilizer, but most of it is fixed in the soil by metal ions such as Ca^2+^, Fe^3+^, Mn^2+^, which in turn cause soil acid-base imbalance and ecological pollution [5]. In addition, phosphate ore, which is used to make phosphate fertilizer, is a non-renewable resource, and, with the increasing agricultural demand for phosphate fertilizer, phosphate ore will face depletion in the next 50 to 100 years [6]. To cope with the future crisis of Pi deficiency in agricultural production, it is, therefore, necessary to gain a deeper understanding of the molecular mechanisms underlying the response of plants to low phosphorus (LP) signals and the regulation of Pi homeostasis.

Plants undergo a series of changes to resist chronic LP stress, including changes in root morphology, root secretions, molecular levels, and metabolic pathways. The root system is the primary organ for nutrient uptake in plants, and the adaptive changes in plant root morphology play an important role in Pi uptake [7]. The roots can adapt to LP stress by inhibiting primary root elongation and promoting the expansion and growth of lateral roots, as well as the expansion of root hairs and the root/shoot ratio [8]. Acid phosphatase (ACP) synthesis and secretion are also increased in plants under LP stress. Polymorphism of *GmACP1*, which encodes acid phosphatase, has been discovered to cause a 33% variation in soybean LP tolerance [9]. In addition, Pi starvation alters the expression of genes related to cell development, hormone metabolism, signaling, and Pi uptake. Bhosale et al. [10] found that auxin, assisted by TRYPTOPHAN AMINOTRANSFERASE OF ARABIDOPSIS1 (*TAA1*, auxin synthesis) and *AUX1* (auxin transport), is transported from the root tip to the differentiation zone to promote root hair growth under Pi deficient conditions. Also, the elevated indoleacetic acid auxin (IAA) levels in root apexes induce transcriptional expression cascades of transcription factors (TFs) ARF19 [11], bHLH families ROOT HAIR DEFECTIVE 6-LIKE 2 (RSL2), and ROOT HAIR DEFECTIVE 6-LIKE 4 (RSL4) to promote root hair elongation [12]. LP conditions promote ethylene (ET) biosynthesis, and ETHYLENE-INSENSITIVE3 (*EIN3*) interacting with the promoters of RSL4 target genes is a key TF for ET signaling [13]. Furthermore, *EIN3* forms complexes with *FHY3*, *FAR1*, and *HY5*, and is bound to the *PHR1* promoter, regulating its expression and driving Pi starvation response (PSR) [14]. Pi homeostasis in plants is also critical in the response to LP stress, and PHOSPHATE TRANSPORTER 1 (PHT1) proteins control Pi uptake and reactivation in plants [15,16]. It has been demonstrated that PHOSPHATE TRANSPORTER TRAFFICFACILITATOR1 (PHF1) controls the intracellular membrane-mediated translocation of PHT1 protein to the plasma membrane and that the phosphorylation of the protein is inhibited under Pi stress, which promotes PHT1 expression [17]. The changes in metabolic pathways are also a response strategy for plant adaptation to stress. LP stress is found to increase the synthesis of sucrose in all Arabidopsis [18], soybean [19], barley [20], and maize [21]. Under Pi deficient levels, plants could enable selective glycolytic pathways, bypassing steps in the glycolytic pathway that require Pi or ATP to safeguard energy production and carbon shelf formation, such as shifting from pathways that require Pi-dependent NAD-G3PDH and phosphoglycerol kinase to pathways that do not depend on the involvement of non-phosphorylated NADP-G3PDH [22].

Transcriptome sequencing technology has been extensively used in crop research, including wheat, rice, maize, and potato, to gain insights into various aspects of plant biology, such as physiological and molecular responses, genome sequencing, gene regulation, gene differentiation, post-transcriptional modifications, and gene splicing [23]. Zhang et al. [24] used the transcriptome to identify 34 root candidate genes and 81 leaf candidate genes under LP stress, and the overexpression of the candidate gene *GmACP2* increased the Pi efficiency of soybean hairy roots by 19.9%, indicating that *GmACP2* plays an important role in LP stress tolerance in soybean. In plant research, the metabolomic analysis could be used to better understand plant responses to stress, especially Pi starvation, and how these responses contribute to the overall plant phenotype, which helps unravel the intricate mechanisms that support plant responses to stress [25]. Unlike conventional chemical analysis, metabolomics use liquid chromatography/mass spectrometry (LC/MS) to detect a wide variety of compounds [26]. Through the identification of various compounds, such as metabolic byproducts and signal transduction molecules under stress conditions, metabolomics can significantly improve plant response to abiotic stress [25]. The combination of the metabolome, transcriptome, and proteome can help to further validate the mechanisms underlying plant responses to abiotic stresses [27,28]. Wang et al. [27] investigated the changes in the gene/metabolite activity of both Dianli-1299 and Dianli-71 through integrated transcriptomic and metabolomic analyses, indicating that key pathways related to glycerophospholipid, glycerolipid, and glycolysis, and glyconeogenesis metabolism play an important role in the regulation of LP stresses tolerance in quinoa seedlings.

LP tolerance in plants is a complex and multi-factored regulatory process. The transcriptional and metabolic mechanisms in wheat under LP stress have not been extensively studied. This study used physiological, transcriptomic, and metabolomic analyses of G28 (LP-tolerant variety) and L143 (LP-sensitive variety) to examine the expression of different genes and the synthesis of different metabolites in wheat roots under Pi starvation treatments. The outcomes indicated that the candidate genes and metabolites in critical biological pathways involved in LP tolerance in wheat could be important regulators. This study provided genetic resources for further discovery of candidate genes for LP tolerance in wheat.

## 2. Results

### 2.1. Phenotypic and Physiological Differences of Two Varieties under LP Stress

**Figure 1:**
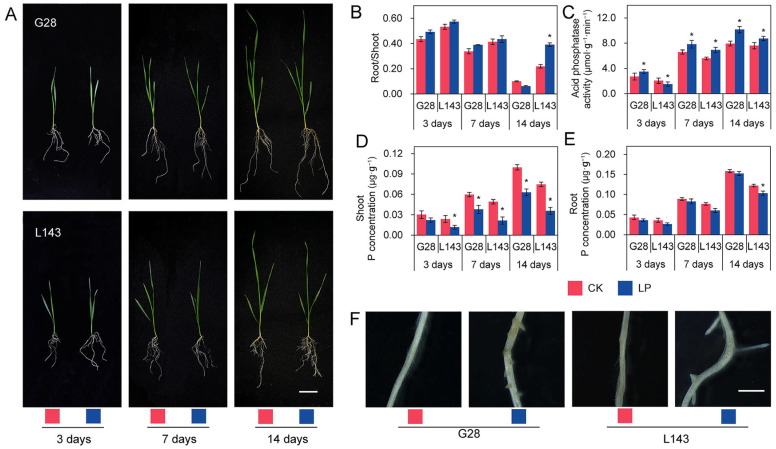
The phenotypic and physiological changes in the G28 and L143 varieties when exposed to LP levels. (**A**) Growth of G28, and L143 under LP stress at different treatment times. Scale bar = 5 cm. (**B**) Root/shoot ratio (%). (**C**) Acid phosphatase activity in roots (μmol·g^−1^·min^−1^). P concentration in shoot (**D**) and root (**E**) (μg·g^−1^). Student’s method was used to compare significant differences between LP and control treatments. * (*p* < 0.05) represents the significance of the difference between two treatments of the same variety at the same time point. (**F**) Lateral rooting protrusions of G28 and L143 were observed after 72 h of treatment with LP. Scale bar = 2 mm. CK—normal phosphorus treatment; LP—low phosphorus treatment.

The phenotypic data revealed that L143 had significant differences, while the roots of G28 were the most developed and had less variation in aboveground growth potential at 14 days of LP stress compared with normal phosphorus (CK) (Figure 1A). Under LP stress, the root/shoot ratio of both varieties increased when compared with CK. While the LP-sensitive variety L143 reached a significant increase of 78.4% (*p* < 0.05) at 14 days of LP stress, the LP-tolerant variety G28 showed less increase at 3, 7, and 14 days of LP stress (Figure 1B). At physiological levels, the ACP activity in root tissues increased with increasing duration under LP stress, although the increase varied between phosphorus utilization efficiency varieties. Under LP stress for 14 days, the increase was smaller in L143, but it was the greatest in G28, increasing 1.28-fold (Figure 1C). Additionally, we observed a significant decrease in the concentration of phosphorus in shoots of L143 as the duration of LP stress increased. The root of L143 showed a significant decrease at 14 days, while the other effects were not significant (Figure 1D,E). These findings showed that L143 was less able to adapt to the changes in external phosphorus levels than G28 under Pi starvation.

To select appropriate samples for sequencing, G28 and L143 were subjected to LP and normal conditions. As shown by a Leica stereomicroscope, after 3 days of LP stress treatment, adventitious root protrusions were more numerous and longer in L143 than in G28 (Figure 1F), showing that L143 roots responded to LP stress more strongly than G28 roots. Thus, wheat roots that had been treated for 3 days were chosen as the source of the metabolomic and transcriptional sequencing samples.

### 2.2. Metabolic Profile of Wheat in Response to LP Stress

**Figure 2:**
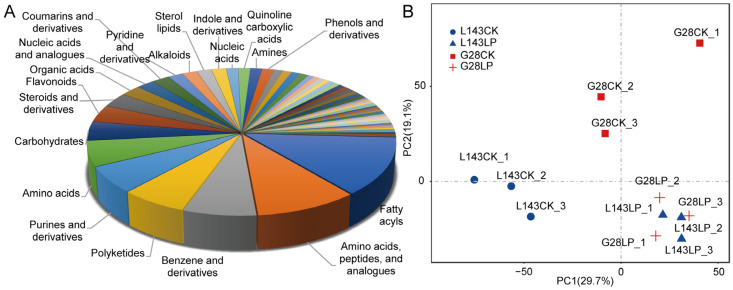
Metabolite profiles identified in G28 and L143 roots with 3 days of treatments involving CK and LP. (**A**) Class of all known metabolites. (**B**) Using metabolomic data, principal component analysis (PCA) was conducted. The first principal component (PC1) is represented by the *X*-axis, and the second principal component is represented by the *Y*-axis (PC2).

To evaluate the systematic profile of metabolic changes in response to LP stress, an untargeted metabolome analysis using LC-MS/MS technology between LP-sensitive (L143) and LP-tolerant (G28) varieties was carried out. A total of 740 of those were recognized metabolites (Table S1). The known metabolites could be divided into 62 categories, such as fatty acyls (13%), polyketides (6%), purines and derivatives (6%), carbohydrates (4%), flavonoids (4%), benzenes and derivatives (7%), steroids and derivatives (3%), amino acids, peptides(10%), organic acids (2%), nucleic acids (1%), etc. (Figure 2A). The principal component analysis of samples provided a preliminary understanding of the overall metabolic differences between the groups. Figure 2B shows that the CK and LP treatments could be distinguished, indicating that there were significant differences in the metabolites of the two treatments.

**Figure 3:**
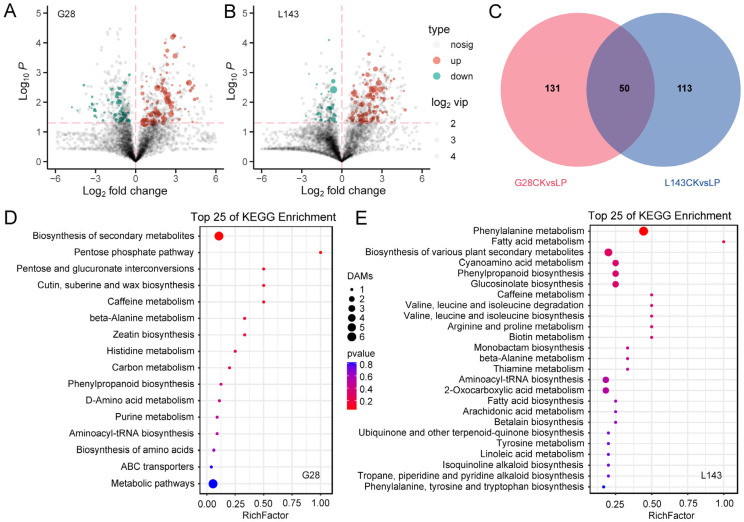
Response of differentially accumulated metabolites (DAMs) in wheat to LP stress. Volcano plots for DAMs with the comparison of G28CK vs. G28LP (**A**) and L143CK vs. L143LP (**B**), the red dotted lines represent the DAMs screening criteria. Venn diagram showing DAMs in roots. (**C**) Top 25 KEGG pathways of DAMs under LP conditions in G28 (**D**) and L143 (**E**). The top 25 statistics of KEGG pathway enrichment are shown. The dot color represents the size of the *p* value. The dot size represents the number of DAMs contained in each pathway.

To explore the metabolomic changes in response to LP stress in wheat, differentially accumulated metabolites (DAMs) were analyzed based on the OPLS-DA model with the screening criteria of VIP ≥ 1 and *t*-test *p* < 0.05. In the comparison groups G28CK vs. G28LP and L143CK vs. L143LP, a total of 181 (113 upregulated; 68 downregulated) and 163 (121 upregulated; 42 downregulated) DAMs were found, respectively. A total of 294 non-redundant DAMs were found to be DAMs connected to the LP response in the wheat root in the two comparison groups (Figure 3A–C and Table S2).

### 2.3. The Response of the Known Metabolites to LP Stress in Wheat Root

The DAMs were identified under LP stress for both varieties (Figure 3A–C and Table S2). 

However, some of these DAMs were known metabolites. The DAMs of G28 and L143 were classified into nine categories, including purines and derivatives, phenylpropanoids, carbohydrates, organic acids, fatty acyls, alkaloids and derivatives, amino acids, peptides, analogues, polyketides, and benzenes and derivatives. Notably, seven metabolites, namely purine (2.908/4.823-fold), 7-methylxanthine (3.095/5.352-fold), 4-morpholinecarboxamide (4.438/6.774-fold), 13(S)-HpOTrE (2.378/4.084-fold), PMK ethyl glycidate (6.498/2.694-fold), ferulic acid (4.627/4.659-fold), and N-{[(2R,4S,5R)-5-Ethyl-1-azabicyclo[2.2.2]oct-2-yl]methyl}-2-propanamine (4.000/2.266-fold), which were common to both the G28 and L143 DAMs, were upregulated under LP stress. Pilocarpine (C07474) significantly downregulated the change 4.77-fold to the G28CK vs. G28LP, whereas neochlorogenic acid (C17147) significantly upregulated the change 2.34-fold to the L143CK vs. L143LP (Table 1). KEGG pathway enrichment analysis was performed to reveal the most relevant pathways associated with the responses of G28 and L143 to LP stress. While the DAMs of G28 were primarily involved in the pentose phosphate pathway, pentose and glucuronate interconversions, cutin, suberin and wax biosynthesis, and histidine metabolism (Figure 3D), the DAMs of L143 were primarily involved in phenylalanine metabolism fatty acid metabolism, cyanoamino acid metabolism, phenylpropanoid biosynthesis, valine, leucine and isoleucine degradation and biosynthesis, and arginine and proline metabolism (Figure 3E).

### 2.4. Transcriptome Identification of DEGs in G28 and L143 under LP Stress

**Figure 4:**
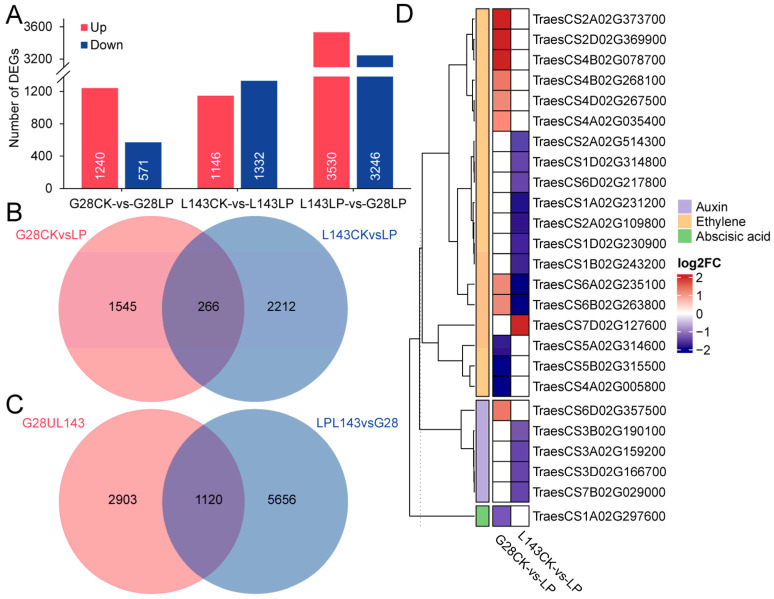
Differentially expressed genes (DEGs) of wheat in response to LP stress. (**A**) Numbers of DEGs under different comparisons. (**B**) Venn diagram illustrating the genes of G28 and L143 in response to LP stress. (**C**) Venn diagram showing the response of DEGs to LP stress and different varieties under LP conditions. (**D**) Heatmap of the DEGs associated with LP stress involved in plant hormone signaling.

To understand how wheat roots responded differently to LP stress at the transcript level, the global transcriptome profiles of G28 and L143 were analyzed. The 551.5 M clean reads totaling 82.72 Gb were produced from 12 samples, with an average GC content of 52.29% and Q20 > 92.72% (Table S3). |log2(Fold change)| ≥ 1 and *p* < 0.05 were used as screening conditions for the DESeq2 analysis. To begin with, we compared the differentially expressed genes (DEGs) of the same variety in different Pi content levels. A total of 1811 DEGs (1240 upregulated; 571 downregulated) and 2478 DEGs (1146 upregulated; 1332 downregulated) were identified in G28CK vs. G28LP and L143CK vs. L143LP, respectively. Meanwhile, a total of 4023 non-redundant DEGs were discovered to be connected to LP response in wheat roots (Figure 4A,B and Table S4). Next, we discovered 6776 DEGs (3530 upregulated; 3246 downregulated) between G28 and L143 under LP conditions (Figure 4B and Table S4). Based on the results of the two partial DEGs mentioned above, it was discovered that the DEGs related to Pi starvation response in wheat roots shared 1120 DEGS with the DEGs in different wheat roots under LP stress (Figure 4C and Table S4). The 1120 DEGs were shared in the two cultivars under LP conditions and connected to the response of wheat root to LP, which might be the key to the difference in LP tolerance of the two cultivars and were considered as the core DEGs of LP tolerance of wheat.

The analysis of DEGs associated with Pi starvation response revealed that, after 3 days of treatment, G28 and L143 had six common DEGs associated with Pi starvation response, including TraesCS4A02G416400 (*PHT1;2*) and TraesCS4B02G317000 (*PHT1;2*), which were upregulated in both G28 and L143. Notably, the upregulated expression fold was higher in L143 (6.404/6.956-fold) than in G28 (3.144/2.097-fold). G28 had 27 specifical DEGs, including 19 upregulated genes such as TraesCS3A02G242700 (LPP2, 13.735-fold) and 8 downregulated genes such as TraesCS4A02G425300 (SPX, 0.477-fold). However, L143 had 35 specifically expressed genes, including 25 downregulated genes such as TraesCS2D02G165100 (UDP-glycosyltransferase 1, 0.307-fold) and 10 upregulated genes such as TraesCS5A02G225600 (WRKY40, 123.607-fold). Notably, TraesCS4A02G416400 (PHO1;2) and TraesCS4B02G317000 (PHO1;2) exhibited exclusive downregulation in the L143 (Table S5). Additionally, 25 DEGs related to phytohormone signaling were identified, including 19 DEGs for ethylene signaling, 5 DEGs for growth hormone signaling, and 1 DEG for ABA signaling (Figure 4D).

### 2.5. GO and KEGG Analysis of DEGs in G28 and L143 under LP Stress

**Figure 5:**
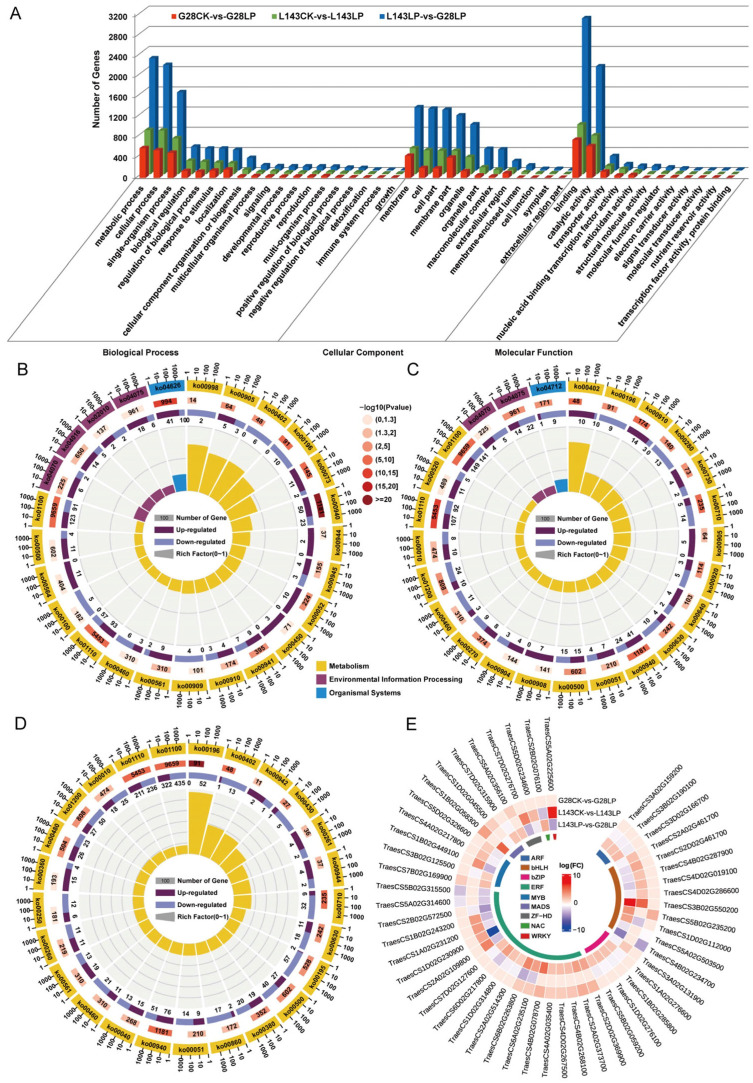
GO, KEGG enrichment, and transcription factors’ (TFs) analysis for all samples of LP resistance traits in wheat. (**A**) GO enrichment analysis based on the differentially expressed genes (DEGs) under different comparisons. KEGG pathway enrichment analysis based on the DEGs in G28CK vs. LP (**B**), L143CK vs. LP (**C**), and L143LP vs. G28LP (**D**). The top 25 statistics of KEGG pathway enrichment are shown. (**E**) Circular heatmap of the 1120 core DEGs associated with LP stress involved in TFs.

To analyze the functions of the DEGs responding to LP between G28 and L143, GO analysis, which revealed GO term enrichment in three major functional categories—molecular function (MF), cellular component (CC), and biological process (BP—was carried out using the enrichGO function in the R packages clusterProfiler. The top 25 enriched GO terms from all DEGs across different comparisons are shown in Table S6. The DEGs between G28CK vs. G28LP and L143LP vs. G28LP were enriched in all three GO categories. In the BP category, the terms oxidative stress and photosynthesis were most enriched, respectively. In the MF categories, the terms antioxidant activity and nucleotide binding were most enriched, respectively. In the CC categories, the terms membrane protein complexes and thylakoid membrane protein complexes were most enriched, respectively (Table S6). The DEGs in the L143CK vs. L143LP were enriched in the BP and MF categories, in which the terms of oxidative stress and antioxidant activity were most enriched (Table S6). Meanwhile, the DEG among G28CK vs. G28LP, L143CK vs. L143LP, and L143LP vs. G28LP were significantly enriched (FDR < 0.05) into 27 functional subgroups, including 16 in the BP, 9 in the MF, and 2 in the CC categories (Figure 5A). In the three comparison groups, the GO term associated with oxidative stress had more upregulated DEGs than downregulated DEGs, whereas the GO term associated with photosynthesis had more downregulated DEGs (Figure 5A).

To further explore the enriched metabolic pathways related to LP stress tolerance and adaptation in wheat, the DEGs from G28CK vs. G28LP, L143CK vs. L143LP, and L143LP vs. G28LP were subjected to KEGG enrichment analysis (Figure 5B–D and Table S7). The top 25 KEGG enrichment pathways detected in G28CK vs. G28LP and L143CK vs. L143LP were classified into three categories, including metabolism, environmental information processing, and organismal systems, whereas in L143LP vs. G28LP, they were grouped into only metabolism (Figure 5B–D). The G28CK vs. G28LP results showed that the secondary metabolite biosynthesis (ko00940, ko00402, ko00905, ko00380), galactose metabolism (ko00052), photosynthesis antenna proteins (ko00196), and phosphatidylinositol signaling system (ko04070) were mostly enriched (Figure 5B). According to the results of L143CK vs. L143LP, the secondary metabolite biosynthesis (ko00940, ko00402, ko00280, ko00730, ko00270, ko00905, ko00460), starch and sucrose metabolism (ko00500), photosynthesis antenna proteins (ko00196), and glycolysis/gluconeogenesis (ko00010) were the most enriched (Figure 5C). Intriguingly, 10 DEGs were upregulated in the L143-enriched benzoxazinoid biosynthesis (ko00402) pathway under LP compared with CK conditions, while all DEGs were downregulated in the G28. The results of L143LP vs. G28LP showed that photosynthesis (ko00195, ko00196), biosynthesis of secondary metabolites (ko00940, ko00402, ko00380, ko00430, ko00460, ko00261, ko00942, ko00260), starch and sucrose metabolism (ko00500), and glycolysis/gluconeogenesis (ko00010) were the most enriched (Figure 5D). Notably, most of the genes in the pathway ko00942, ko00261, ko00944, and ko00940 enriched by the L143LP vs. G28LP comparison group were upregulated DEGs.

### 2.6. Identification and Analysis of TFs for Core DEGs

To date, a sizable number of TFs linked to LP-inducible expression genes have been isolated and verified in plants. Thus, the TFs’ identification and analysis of 1120 core DEGs showed that a total of 73 significantly different TFs were identified in regulated wheat LP tolerance between G28 and L143 and divided into 22 categories (Figure S2). The expression levels of TFs related to plant LP resistance were analyzed, and the results showed the differential gene expression was significant in bHLH, ARF, ERF, and MYB (Figure S2). Notably, we found that 60% (6/10) of bHLH family TFs were upregulated in the L143CK vs. L143LP comparison, and only 10% (1/10) of bHLH family TFs were upregulated in the G28CK vs. G28LP comparison (Figure 5E). These results indicated the different responses to Pi starvation between G28 and L143 at transcriptomic levels.

### 2.7. Integrated Transcriptomic and Metabolomic Analyses

**Figure 6:**
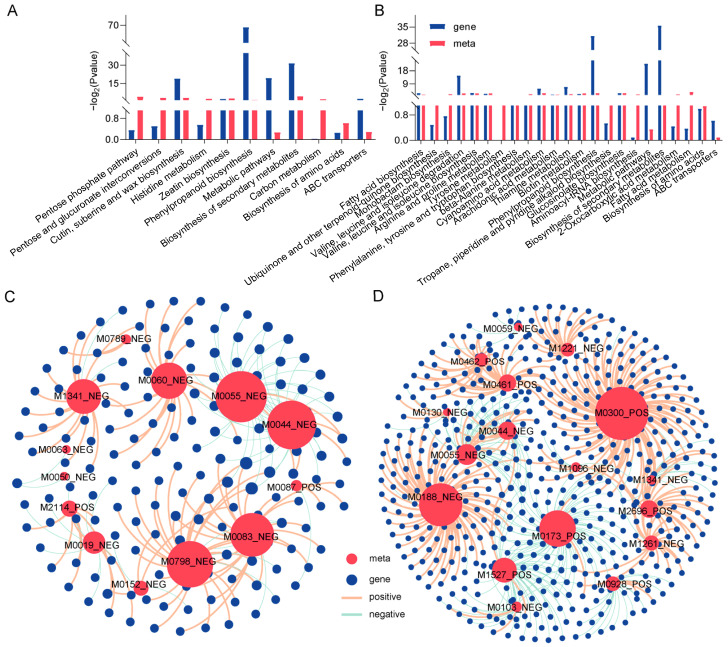
Transcriptome and metabolome-combined KEGG enrichment and correlation network analysis. Joint KEGG enrichment of histograms for G28CK vs. LP (**A**) and L143CK vs. LP (**B**). Correlation network analysis of DAMs and DEGs related to LP tolerance in wheat for G28CK vs. LP (**C**) and L143CK vs. LP (**D**). Metabolites are marked red, genes are blue. The red lines indicate a positive correlation; the green lines indicate a negative correlation. The size of the red circle represents the number of genes associated with the metabolite. The line thickness between nodes represents the degree of correlation between two nodes.

The integration of metabolomics and transcriptomic data further revealed that G28 and L143 responded to LP stress. Under Pi starvation stress, DAMs and DEGS of G28 were shown to be enriched in the same KEGG pathway, which was primarily connected to the pentose phosphate pathway, pentose and glucuronate interconversions, cutin, suberin and wax biosynthesis, histidine metabolism, zeatin biosynthesis, phenylpropanoid biosynthesis, and the biosynthesis of amino acids (Figure 6A), whereas L143 was primarily connected with fatty acid biosynthesis, valine, leucine and isoleucine biosynthesis, arginine and proline metabolism, tyrosine metabolism, phenylalanine, tyrosine and tryptophan biosynthesis, and phenylpropanoid biosynthesis, implying that the wheat roots might alleviate LP stress via a series of energy metabolism and amino acid biosynthesis mechanisms (Figure 6B).

Gene–metabolite interaction networks could help understand functional relationships and identify novel regulatory elements. Pearson correlation analysis was performed for DEGs and DAMs of G28 and L143, respectively. In G28 (Figure 6C), 105 DEGs were strongly positively correlated with 11 metabolites (r^2^ > 0.9, *p* < 0.001), and 57 DEGs were strongly negatively correlated with 10 metabolites (r^2^ < −0.9, *p* < 0.001). Further analysis revealed that the expression of most DEGs in G28 (such as TraesCS2A02G046600, TraesCS3A02G246200, and TraesCS5B02G382400) was associated with gluconic acid lactone (M0060_NEG), fumaric acid (M0083_NEG), alpha-eleostearic acid (M0798_NEG), ferulic acid (M1341_NEG), and four key metabolites were significantly and positively correlated with the expression of most DEGs (such as TraesCS2B02G241400, TraesCS2D02G585200, TraesCS4A02G342500, etc.). The two DAMs that were negatively correlated with the expression of most DEGs (such as TraesCS2B02G241400, TraesCS2D02G585200, and TraesCS4A02G342500) were purine (M0044_NEG) and 7-methylxanthine (M0055_NEG). In L143 (Figure 6D), the expression of most DEGs (such as TraesCS4D02G329500, TraesCS1B02G075200, TraesCS5D02G312100, etc.) was significantly and positively correlated with the expression of L-isoleucine (M0300_POS) and L-tyrosine (M0188_ NEG). Two key metabolites were significantly and positively correlated with the expression of most DEGs (such as TraesCS3D02G439400, TraesCS3A02G001700, TraesCS4A02G200200, etc.). The two DAMs that were negatively correlated with the expression of most DEGs (such as TraesCS3D02G439400, TraesCS3A02G001700, TraesCS4A02G200200, etc.) were 4-guanidinobutyric acid (M0173_POS) and 9-KODE (M1527_POS).

**Figure 7:**
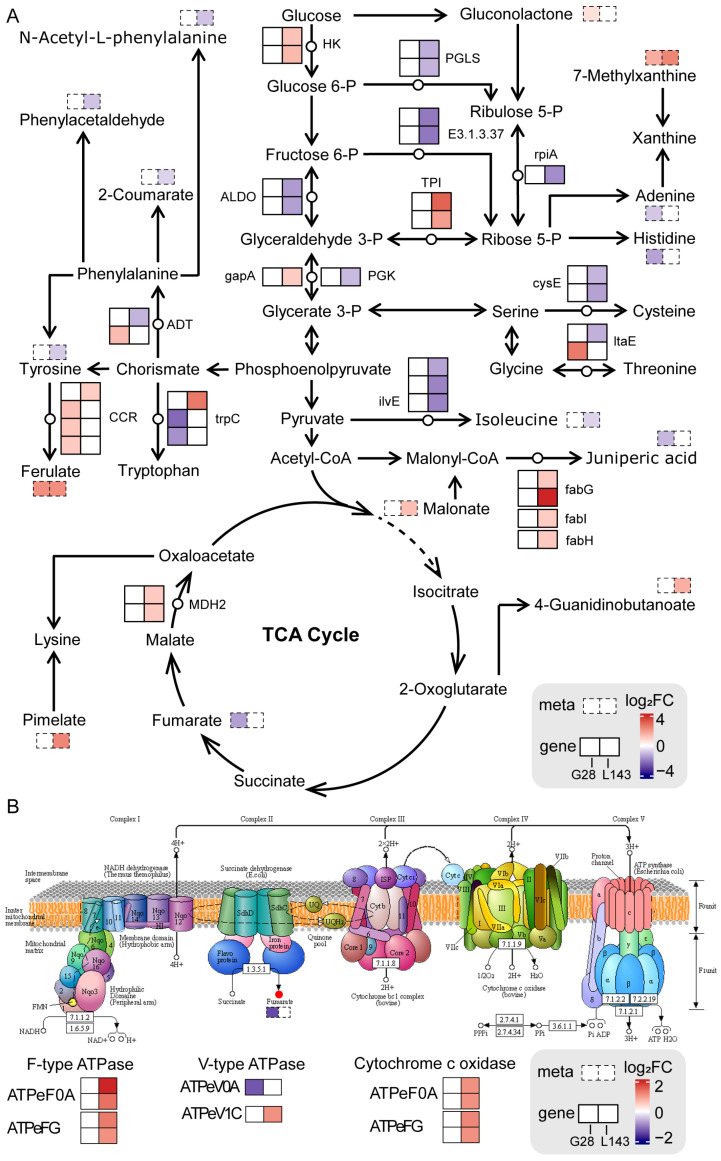
Response mechanisms of DEGs and DAMs in the sugar and amino acid metabolism (**A**) and oxidative phosphorylation (**B**) pathways in wheat roots under LP stress. DEGs and DAMs are represented by the solid line and dotted line boxes in pathways, respectively. Red and blue represent upregulated and downregulated genes/metabolites, respectively. HK—hexokinase; PGLS—6-phosphogluconolactonase; rpiA—ribose 5-phosphate isomerase A; E3.1.3.37—sedoheptulose-bisphosphatase; ALDO—fructose-bisphosphate aldolase, class I; TPI—triosephosphate isomerase; gapA—glyceraldehyde 3-phosphate dehydrogenase; PGK—phosphoglycerate kinase; MDH2—malate dehydrogenase; cysE—serine O-acetyltransferase; fabG—3-oxoacyl-[acyl-carrier protein] reductase; fabI—enoyl-[acyl-carrier protein] reductase I; fabH—3-oxoacyl-[acyl-carrier-protein] synthase III; ilvE—branched-chain amino acid aminotransferase; ltaE—threonine aldolase; CCR—cinnamoyl-CoA reductase; trpC—indole-3-glycerol phosphate synthase; ADT—arogenate/prephenate dehydratase.

To comprehensively assess the potential molecular mechanisms of LP stress in both varieties, we mapped comprehensive systems biology pathways by analyzing the KEGG pathways of DEGs and DAMs (Figure 7A). We noticed that ferulate and 7-methylxanthine accumulated identically in both genotypes. The accumulation of N-acetyl-L-phenylalanine, phenylacetaldehyde, 2-coumarate, tyrosine, and isoleucine was reduced in L143, while the difference was not significant in G28. Also, more DEGs were significantly upregulated in L143 compared with G28. The results above suggested that the expression of specific genes and the accumulation of metabolites under the same metabolic pathway, as well as the regulation of specific metabolic pathways, led to the differences in the response to low phosphorus stress in the two cultivars.

### 2.8. qRT-PCR Verification of RNA-Seq Data

We selected 15 DEGs associated with response to LP stress for qRT-PCR analysis to validate the RNA-seq data. When these genes were treated accordingly, the qRT-PCR analysis showed that the expression patterns of these genes were closely correlated with the FPKM values obtained by sequencing. These findings suggested that the data from RNA-seq were reliable (Figure S3).

## 3. Discussion

Plants responded to LP through a series of multifaceted morphological, physiological, metabolic, biochemical, and molecular changes, collectively referred to as PSR, which might improve Pi acquisition, Pi redistribution and transport, and overall Pi use efficiency [29]. Plants typically increased their root/shoot ratio by reducing stem growth more than root growth, as well as increasing the number and length of lateral roots and root hairs when Pi became restricted [8]. In the present research, similar results were also found (Figure 1A,B,D). Also, plants under LP stress considerably increased production and secretion of ACP, which would be helpful to the release, removal, and recycling of Pi from the organic materials in the soil and within the plant [30]. The ACP activity in the roots of the LP-tolerant variety G28 in this study increased more than that of the LP-sensitive variety L143(Figure 1C), indicating that low phosphorus stress had different regulatory effects on the ACP activity in the roots of different LP-tolerant wheat and, consequently, their ability to reuse Pi differently. Accordingly, we further investigated the changes in genes and metabolites caused by Pi starvation stress in two wheat roots with varying LP tolerance.

### 3.1. Signaling in Wheat Root under Pi Starvation

To date, a sizable number of TFs linked to LP-inducible expression genes have been isolated and verified in plants. Thus, the TFs’ identification and analysis of 1120 core DEGs showed that a total of 73 significantly different TFs were identified in regulated wheat LP tolerance between G28 and L143 and divided into 22 categories (Figure S2). The expression levels of TFs related to plant LP resistance were analyzed, and the results showed the differential gene expression was significant in bHLH, ARF, ERF, and MYB (Figure S2). Notably, we found that 60% (6/10) of bHLH family TFs were upregulated in the L143CK vs. L143LP comparison, and only 10% (1/10) of bHLH family TFs were upregulated in the G28CK vs. G28LP comparison (Figure 5E). These results indicated the different responses to Pi starvation between G28 and L143 at transcriptomic levels.

After sensing Pi starvation stress in the roots or elsewhere in the plant, signaling pathways/cascades were activated, leading to the generation of local and systemic signals [31]. Pi deficiency could change the expression of hormone biosynthetic genes, hormone production, sensitivity, signaling, and transport to control root growth, including the inhibition of primary root growth, the promotion of lateral root growth, root-hair, and cluster-root formation [32]. The present study found that most of the auxin, ethylene, and ABA-related genes were downregulated, indicating that the response of wheat roots to LP stress was significantly influenced by the control of gene expression of related hormones (Figure 4D). During Pi starvation, auxin synthesis, transport, and signaling played important roles in the regulation of root growth and architecture [29]. In Arabidopsis Pi deficient roots, auxin sensitivity was believed to be enhanced by the increased expression of the auxin receptor TIR1, leading to the degradation of the AUX/IAA response repressor and the ARF TFs becoming active and activating/repressing genes involved in lateral root formation and emergence [33]. ARF TFs in rice were also associated with regulating PSRs [11]. We discovered that the expression of L143 auxin-related genes (ARFA and IAA20) was downregulated in response to low phosphorus stress, whereas IAA9 was upregulated in G28 (Figure 4D), indicating that the auxin signaling response pattern of L143 under LP stress differed from that of G28, with faster differential expression of L143 auxin signaling-related genes allowing it to regulate root development more rapidly in response to LP stress (Figure 1F). ET also played a role in regulating the plant response to Pi starvation. It was found that ET levels changed under phosphorus stress and regulated the growth of lateral roots and root hairs [13]. Increased ET levels could promote root cell senescence and the formation of aeration tissue [34]. Transcriptome analysis revealed that the expression of ERF1, ERF2, and ERF5 was significantly altered in Pi starvation-induced Arabidopsis roots [35,36]. In double mutants of PSRs factors, the expression of at least eight AP2/ERF genes was significantly reduced [37]. Our results revealed that the expression of ERF53, ERN1, and RAP2-4, genes related to ET sensing, were downregulated in G28 under LP treatment, whereas L143 was upregulated with the expression of ERF53, ERF54, ERF78, RAP2-3, and RAP2-10 genes related to ET sensing (Figure 4D), suggesting that ET might play an important role in G28 and L143 root systems in response to LP stress.

Similarly, TFs are very important regulatory nodes in the regulatory network of plant LP stress response. It has been demonstrated that both MYB2 and MYB62 could be involved in the Pi deficiency response in *Arabidopsis*, with MYB2 acting as an activator to directly regulate the expression of AtmiR399f, which in turn promoted *Arabidopsis* adaptation to Pi-deprived context, whereas MYB62 was negatively involved in the LP-induced regulation, primarily by the gibberellin synthesis and signaling pathway [38]. In addition, another MYB-CC structural domain-containing TF was also involved in the regulation of Pi deprivation response in plants, such as AtPHR1, OsPHR1, OsPHR2, OsPHR3, OsPHR4, and TaPHR1 [39]. After the knockdown of the *TaPHR3-A1* gene, wheat exhibited retarded seedling growth and root hair development, and presented significant yield-related effects under LP and Pi-abundant conditions at maturity [40]. Five MYB-related genes were identified in this study, with MYB7 in G28 and MYB4, MYB85, and RAD-like 6 in L143 being upregulated, whereas the MYB-related gene was mostly downregulated expression in L143 (Figure 5E). Hence, we speculated that MYB might have more complex and diverse regulatory patterns in wheat roots in response to LP stress. *AtWRKY6*, a gene related to the WRKY TF, was discovered during the induction of LP in *Arabidopsis*. Functional analysis revealed that *AtWRKY6* could be bound to the W-box of the promoter of the *AtPHO1* gene, which in turn was involved in the reaction to LP stress [41]. The overexpression of the rice WRKY III subfamily gene *OsWRKY74* improves plant tolerance to LP and increases biomass and Pi content by stimulating the expression of LP-responsive genes; in addition, *OsWRKY74* is implicated in plant response to Fe and cold damage [42]. *WRKY40* (TraesCS5A02G225600) was significantly upregulated in L143 (123.6-fold) but not differentially expressed in G28 (Figure 5E). The results indicated that the LP-sensitive material L143 might have promoted the development of L143 root projections for rapid adaptation to LP stress by the induction of WRKY expression. Other than MYB and WRKY, TFs such as bHLH and MADS have been discovered to have active roles in plant response to LP stress. For instance, rice *OsPTF1* encoded a bHLH TF that positively regulated root conformation (root length and root area), increased plant Pi concentration, and enhanced LP tolerance [43]. *ZmPTF1* was an early documented TF involved in Pi stress response in maize, which was significantly induced by LP to promote root formation, improve plant tolerance to Pi deficiency, and boost maize yield under LP circumstances [44]. Han et al. [45] revealed that the Mα-like MADS-box gene *TaMADS2* was involved in activating Pi response signals that regulated root growth and its response to LP stress. The present study identified 10 bHLH and 3 MADS TF-related genes, of which bHLH was primarily upregulated in L143, while MADS was mostly upregulated in G28 (Figure 5E).

### 3.2. Pi Transporters Play Essential Role in Pi Homeostasis in Wheat under LP Stress

Pi transport proteins played a role in the process by which plant roots absorbed Pi from the soil and delivered it to different tissues and organs for utilization [15]. Based on the location and functional structure of PHT proteins, they could be broadly classified into four categories, namely PHT1, PHT2, PHT3, and PHT4 [46], among which PHT1 had the strongest transport activity and capacity. The activity and capacity might be owing to PHT1 location on the plasma membrane of plant cells and the ability to more easily cross the cell membrane to achieve Pi transport [46]. Similarly, PHO1 plays an important role in root-to-stem Pi transport [46]. In this study, TraesCS4A02G416400 and TraesCS4B02G317000, which belonged to the PHT1 family, were both upregulated in wheat roots under Pi deficiency levels (with L143 upregulated expression fold higher than G28), while *PHO1* genes (TraesCS4A02G416400 and TraesCS4B02G317000) exhibited a specific and significant downregulation in L143 (Table S5). These findings suggest that L143 might experience impaired transport of P from root-to-shoot under LP stress because of the specific downregulation of PhO1;2 expression, which aligns with our observation of P concentrations in both shoots and roots of L143. The transcription levels of plant PHT1 family genes were regulated by several factors, such as the concentration of inorganic phosphorus in the external environment, and ABI [47], MYB [38,40], and WRKY [48] family TFs. All these TFs could be bound to specific regulatory elements in the promoter regions of PHT1 family genes to regulate gene transcription. Zhang et al. [47] showed that ABI promoted Pi acquisition by plants by directly binding the promoter of the *PHT1;1* gene and activating the expression of the latter. The overexpression of *PHT1;1* was able to completely complement the reduced Pi-containing phenotype of the *abi* mutant under LP conditions. We found a significant upregulation of ABI (TraesCS7D02G127600) expression (16-fold) in the LP-sensitive genotype L143 under insufficient Pi supply stress, whereas the expression was not significant in the LP-tolerant genotype G28 (Figure 4D). This result suggested that ABI in L143 under LP treatment might positively regulate the expression of PHT1, which in turn promoted root acquisition for Pi. *AtPHR1* was the first MYB superfamily TF isolated in vascular plants involved in the transcriptional regulation of LP stress [38]. The results of the study on the wheat PHR1 homolog *Ta-PHR1-A1* showed that it could activate the expression of *Ta-PHT1.2* [40]. Similarly, the WRKY TF family of genes also played an important role in regulating PHT1 transcription by binding to W-box elements in the promoter region of genes to regulate gene transcription. Wang et al. [48] found *Arabidopsis WRKY45* could bind two W-boxes in the promoter region of *PHT1;1*, thus activating *PHT1;1* gene transcription. In our study, *MYB4*, *MYB85*, *RAD-like 6*, and *WRKY40* were all upregulated in L143 (Figure 5E), indicating that the upregulated expression of MYB and WRKY family-related genes in L143 roots further activated the expression of PHT1 in response to LP stress. Consequently, Pi transporters were important for Pi homeostasis in wheat under Pi starvation stress, especially for the LP-sensitive genotype L143.

### 3.3. Sugar and Amino Acid Metabolism Were Significant for LP Response in Wheat

Not only did sugar supply the carbon needed for plant morphogenesis and energy, but it also acted as a signaling molecule in plants that helped regulate plant growth, development, and stress response, as well as interacting with nutrients such as nitrogen and phosphorus [49]. Sugar transport in siliques during Pi deficiency might be involved in metabolic pathways such as low Pi signaling and Pi transport, root growth, root hair production, ACP secretion, and auxin synthesis [50]. Under Pi starvation, plants could reduce Pi consumption by adjusting glycolytic pathways and transmitting LP stress signals by changing sugar concentration, thereby improving plant adaptation to LP adversity [51]. Previous studies have shown that LP stress and sugar signaling interacted with each other [52]. Relative concentrations of metabolites such as 6-fructose phosphate, 6-glucose phosphate, and inositol phosphate were dramatically reduced in both leaves and the roots of severely Pi deficient plants, while glycerol 3-phosphate in leaves and roots of severely Pi deficient plants showed similar results [53]. This suggested that plants grown in LP environments could recover Pi from low molecular weight phosphorylated metabolites to maintain critical cellular functions [53]. We found that the expression levels of most genes related to the regulation of glycolysis pathway were significantly downregulated under LP stress in L143 (Figure 7A), whereas there was no change in G28. In addition, gluconolactone content was significantly increased in G28. Hence, we hypothesized that LP stress reduced the content of related metabolites in glycolysis by the regulation of related genes, while affecting the root growth of L143 and G28 genotypes.

Also, the key metabolites of glycolysis were involved in the synthesis of energetic substances such as nucleotides and amino acids [54]. However, under Pi-limited conditions, Pi could not be readily used for energy metabolism without its involvement in providing carbon metabolites for the TCA cycle. So, organic acids and amino acids would be the preferred forms of carbon storage for sustained plant growth under LP conditions [54]. Plants grown under Pi-limited conditions must alter metabolic processes to consume available amino acids. Several amino acids would serve as supplemental carbon sources to produce energy, reassimilate excreted ammonium, and produce manganate secretion. Amino acids such as glycine, serine, aminobutyric acid, and glutamine increased in LP-tolerant maize under Pi-deficient stress, while isoleucine, aspartic acid, and phenylalanine levels decreased [53]. In our study, the reduced levels of amino acids and amino acid derivatives such as isoleucine, histidine, serine, and juniperic acid, as well as the related genes (*cysE*, *ilvE*, *ADT*, *trpC,* and *fab*) involved in LP stress mostly showed downregulated expression, especially in the roots of LP-sensitive wheat genotype (L143) grown under severe Pi-limiting conditions with significantly low isoleucine and histidine contents and significantly downregulated expression of *cysE* and *ilvE* (Figure 7A). This suggested that Pi non-supply in the roots of LP-sensitive wheat was more pronounced than in the Pi-deficient plants of the tolerant G28 genotype. These results were similar to former studies.

### 3.4. Oxidative Phosphorylation Was Critical under LP Stress in Wheat

Adenylate and Pi-independent alternative mitochondrial bypass pathways contributed significantly to metabolic reprogramming in LP plants [55]. Long-term Pi deficiency, significant reductions in cytoplasmic ADP, and Pi pools limited oxidative phosphorylation, leading to the inhibition of O_2_ consumption by cytochrome oxidase and cytochrome oxidase coupled with ATP synthesis [56]. In that case, the plant might avoid damage due to blocked electron transport by two non-phosphorylated pathways (the anti-cyanogenic pathway and the bypass of the rotenone insensitive complex I). Even though this respiration was ineffective and produced no energy, it was critical for plant survival [57]. In addition, it was also shown that phosphorylation and the succinylation of proteins in barley during Pi starvation and Pi recovery were involved in oxidative phosphorylation metabolism coupled with ATP synthesis via an electrochemical transmembrane gradient [58]. The increased expression of ATPase subunits was connected to a greater necessity for ATP synthase to export protons from the cell [59]. The combined analysis of DEGs and DAMs showed that the oxidative phosphorylation pathway was enriched for two F-type ATPases genes (*ATPeF0A*, *ATPeFG*), two V-type ATPases genes (*ATPeV1C*, *ATPeV0A*), two cytochrome c reductase genes (*COX5B*, *COX11*) and fumaric acid under LP treatment, except that *ATPeV0A* were expressed upregulated in L143, while fumaric acid content was significantly reduced in G28 roots (Figure 7B). It indicated that LP-sensitive variety L143 might respond positively to Pi deficient stress by regulating the expression of related genes in the oxidative phosphorylation pathway and thus maintaining its survival.

## 4. Materials and Methods

### 4.1. Materials and Sample Preparation

In this study, cultivated wheat G28 (LP-tolerant) and L143 (LP-sensitive), which were screened based on the laboratory’s comparative analysis of LP-tolerance in 162 wheat varieties at the seedling and planting stages, were provided by State Key Lab of Aridland Crop Science/Gansu Key Lab of Crop Improvement and Germplasm Enhancement. The field performance of these two varieties was illustrated in Supplementary Figure S1.

Full and uniformly sized seeds were selected, which were surface-disinfected with 3% NaClO for 10 min, rinsed with distilled water, and germinated in a transparent plastic germinator (12 cm × 12 cm × 5.5 cm) lined with two layers of wet filter paper. After 5 days, seedlings with uniform growth were selected and transferred to hydroponic tanks containing 10 L (1/2 Hoagland) nutrient solution, 24 seedlings per pot, and incubated for 2 days followed by normal phosphorus treatment (0.2 mmol·L^−1^, CK) and low phosphorus treatment (0.02 mmol·L^−1^, LP) with five replications, using KH_2_PO_4_ as the Pi source, while 0.18 mmol·L^−1^ KCl was added to the nutrient solution for LP stress to supplement elemental K. The pH value was controlled in the range of 6.8 ± 0.1, and the rest of the nutrient solution components were the same. The hydroponic experiments were conducted in an artificial climate chamber and the growth conditions were referred to Ren et al. [20]. Wheat seedlings were counted at 0 d from the beginning of the stress treatment and selected at 3 d, 7 d, and 14 d of stress in each treatment environment for phenotypic and physiological data determination. All samples were immediately frozen in liquid nitrogen and stored at −80 °C for transcriptomic and metabolomic analyses.

### 4.2. Phenotypic Physiology and Phosphorus Concentration

A Canon camera (Canon EOS 5D Mark II) was used to take photographs of seedling morphology. Root traits under different phosphorus treatments were observed and photographed with a stereomicroscope (LEICAM165 C; LEICA, Wetzlar, Germany). Five biological replicates were available for each treatment. The root-to-shoot ratio was calculated after drying fresh roots and shoots in an oven at 105 °C to a constant weight. The formula was: root/shoot = root DW/shoot DW. ACP activity was determined using the Plant ACP ELISA kit (Shanghai, China, QC11252). The dried samples were powdered and digested with concentrated H_2_SO_4_ and H_2_O_2_ following the method described by Zhang [9] in order to facilitate the determination of phosphorus concentration with the molybdate-blue colorimetric method.

### 4.3. Transcriptome Sequencing and Metabolome Detection

There were three biological replicates for each group of transcriptome and metabolome sequencing. The Total RNAprep Pure Plant Kit from TIANGEN BIOTECH (Beijing, China) was utilized. Gel electrophoresis and NanoDrop spectrophotometer (Thermo Fisher Scientific, Wilmington, DE, USA) were used to assess quality and purity of RNA. Oligo dT Selection was used to enrich the mRNAs and fragmented by adding interrupting reagent to prepare the cDNA library, and there was paired-end sequencing by the DNBSEQ platform in BGI Genomics Co., Ltd. (Shenzhen, China), with a read length of 150 bp.

A Waters 2D UPLC (waters, Milford, MA, USA) tandem with a Q Exactive high-resolution mass spectrometer (Thermo Fisher Scientific, Wilmington, DE, USA) was used for metabolite separation and detection. The gradient elution was performed using aqueous solution containing 0.1% formic acid and 100% acetonitrile containing 0.1% formic acid at a flow rate of 0.3 mL·min^−1^ and an injection volume of 5 μL. The mass spectrometry scanning was performed in the range of m/z ratios of 150 to 1500. Stepped nce was set to 20, 40, and 60 eV. Electron spray ionization (ESI) parameter settings were as follows: the sheath gas flow rate was set to 40, the aux gas flow rate was set to 10, and the spray voltage was set to 3.80 kV for positive ion mode and 3.20 kV for negative ion mode.

### 4.4. Bioinformatics Analysis

The sequenced raw data in FASTQ format from the current trial were submitted to the SRA (https://www.ncbi.nlm.nih.gov/sra/, accessed on 13 December 2022) with the accession numbers PRJNA911470. Transcriptome sequencing data were filtered with SOAPnuke (v1.5.2) software. All downstream analyses were based on high-quality clean data. The reference accession, the Chinese Spring (bread wheat) genome sequences, and annotation files were downloaded from the IWGSC RefSeq v1.1 database (https://urgi.versailles.inra.fr/download/iwgsc/IWGSC_RefSeq_Annotations/v1.1/, accessed on 10 June 2022). The clean reads were mapped to the reference genome using HISAT2 (v2.0.4). Bowtie2 (v2.2.5) was applied to align the clean reads to the reference. Then, the FPKM of each gene was calculated based on the length of the gene and read count mapped to this gene. The differential expression analysis of two groups (with three biological replicates per condition) was performed using the DESeq2 (1.18.0). Genes with *p* < 0.05 and fold changes > 2 were considered DEGs. ClusterProfiler (3.4.4) software was used to achieve GO enrichment analysis of DEGs. The metabolites were annotated using the KEGG database (http://www.genome.jp/kegg/, accessed on 21 September 2022), and HMDB database (http://www.hmdb.ca/, accessed on 15 September 2022). Orthogonal projection to latent structures/discriminant analysis (OPLS-DA) was applied in comparison groups using R package models. The metabolites with VIP > 1 and *p* < 0.05 were considered to be differential metabolites. Volcano plots, cluster heatmap, and correlation analyses were performed by R (4.1.3). We used the KOBAS software 3.0 to test the statistical enrichment of differentially expressed genes and differential metabolites in KEGG pathways. The KEGG pathways with a corrected *p* value of less than 0.05 were considered significantly enriched by differentially expressed genes. Gene—metabolite interaction networks were visualized using Gephi (v0.9.7) software.

### 4.5. Validation of Quantitative Real-Time PCR (qRT-PCR)

We selected 15 DEGs associated with response to LP stress to verify the RNA-seq data by qRT-PCR. Primers were designed by using Primer Express 3.0.1 and are listed in Table S8. Total RNA from roots at different treatments was extracted and carried out by Rnase-Free Dnase I digestion (TIANGEN, Beijing, China) to remove DNA. The qRT-PCR (20 μL reaction volume) was performed with 10 μL of SYBR Premix Ex Taq Kit (TaKaRa, Shiga, Japan), 2 μL of cDNA, 1.6 μL of primer mix, 0.4 μL ROX Reference Dye (TaKaRa, Shiga, Japan), and 6 μL double-distilled H_2_O on a QuantStudio 5 Real-Time PCR System (Applied Biosystems, Waltham, MA, USA). The gene expression level was calculated by the 2^−ΔΔCt^ method using *TaActin* (accession number: XM_044554036.1) as an internal reference gene.

### 4.6. Statistical Analysis

All data analyses and graphics drawings were accomplished using GraphPad Prism 9 (https://www.graphpad.com/, accessed on 26 May 2023). The statistical significance was calculated by Student’s test. Error bars represented SE (*n* = 5). The difference was statistically significant *p* < 0.05. The experiments were performed with five biological replicates, and plant materials from three seedlings were pooled for each biological replicate.

## 5. Conclusions

The present study conducted a comprehensive analysis of the morphological physiology, transcriptomics, and metabolomics of the root of two different LP-tolerant wheat seedlings. It was found that G28 enhances phosphorus absorption and transportation by increasing root length, promoting acidic phosphatase secretion, upregulating genes associated with auxin (*IAA9*) and phosphorus transporters (*PHT1;2*), and increasing the levels of DAMs in the sugar and amino acid metabolic pathways (gluconolactone and ferulate). These mechanisms help maintain stable phosphorus levels and enhance adaptation to LP stress. Furthermore, MYB4, MYB85, and WRKY40 were identified as important TFs in the LP stress response of the LP-sensitive variety L143. In summary, the variation in tolerance to LP levels between the two wheat varieties was regulated through a complex interplay of plant signal transduction, energy metabolism, and amino acid metabolism pathways. These findings lay the foundation for further elucidating the molecular mechanisms of wheat in response to LP stress.

## Figures and Tables

**Table 1:** Information about significant known DAMs with their log_2_FC values, compound ID, and types of regulations according to the OPLS-DA model.

Metabolite ID	Metabolite Name	Compound ID	VIP Values	log_2_FC	*p*-Value
**8 common known DAMs in G28CK vs. G28LP and L143CK vs. L143LP**					
M0044_NEG	Purine	C00465	5.74/6.52	1.54/2.27	0.022/0.004
M0055_NEG	7-methylxanthine	C16353	5.72/6.55	1.63/2.42	0.024/0.004
M0563_NEG	4-morpholinecarboxamide	-	5.06/4.91	2.15/2.76	0.004/0.000
M0615_NEG	13(S)-HpOTrE	-	1.41/2.03	1.25/2.03	0.022/0.000
M0631_POS	PMK ethyl glycidate	-	1.62/1.19	2.70/1.43	0.019/0.022
M1341_NEG	Ferulic acid	C01494	1.57/1.59	2.21/2.22	0.000/0.004
M1560_POS	N-{[(2R,4S,5R)-5-ethyl-1-azabicyclo[2.2.2]oct-2-yl]methyl}-2-propanamine	-	1.93/1.43	2.00/1.18	0.003/0.007
M2125_POS	4-amino-3-hydroxybenzoic acid	-	1.64/1.20	−1.29/−1.25	0.009/0.045
**27 known DAMs in G28CK vs. G28LP**					
M0019_NEG	L-histidine	C00135	2.16	−1.37	0.0050
M0043_NEG	beta-cellobiose	-	3.35	2.07	0.0490
M0050_NEG	D-lyxose	C00476	14.22	0.67	0.0478
M0060_NEG	Gluconic acid lactone	C00198	1.38	0.59	0.0077
M0063_NEG	Chlorhexidine gluconate	C08038	14.26	0.67	0.0482
M0083_NEG	Fumaric acid	C00122	2.04	−1.42	0.0062
M0087_POS	Griseofulvin	C06686	1.41	−1.27	0.0484
M0094_POS	N3,N4-dimethylarginine	-	1.28	−1.44	0.0376
M0152_NEG	Adenine	C00147	1.27	−0.82	0.0082
M0597_NEG	15-deoxy-delta12,14-prostaglandin J2	-	1.03	2.55	0.0048
M0632_POS	N4-acetylsulfamethazine	-	1.23	2.74	0.0241
M0662_NEG	Kukoamine B	-	1.26	2.16	0.0258
M0789_NEG	16-Hydroxyhexadecanoic acid	C18218	3.99	−1.06	0.0374
M0798_NEG	alpha-eleostearic acid	C08315	1.31	−0.82	0.0267
M0877_NEG	Alisol A 24-acetate	-	1.24	−1.53	0.0390
M0917_POS	Farnesyl acetate	-	1.01	−1.70	0.0034
M1122_NEG	N-acetyl-d-alloisoleucine	-	1.84	−0.82	0.0306
M1134_POS	5-tetradecyloxy-2-furonic acid	-	1.21	2.01	0.0040
M1697_POS	13,14-dihydro-15(R)-prostaglandin E1	-	1.08	3.31	0.0028
M1783_POS	Poly THF n6	-	1.46	−3.07	0.0173
M1993_POS	Monoolein	-	1.21	0.82	0.0291
M2114_POS	Pilocarpine	C07474	1.18	−4.77	0.0148
M2597_POS	Acetic acid	-	1.37	−1.09	0.0444
M2701_POS	1-piperazinecarboxylic acid	-	1.72	−1.23	0.0035
M3152_POS	Benzoic acid	-	2.27	2.83	0.0001
M3180_POS	4-methylumbelliferyl α-D-glucoside	-	1.21	1.38	0.0405
M3317_POS	Di-4-coumaroylputrescine	-	1.78	3.89	0.0023
**27 known DAMs in L143CK vs. L143LP**					
M0059_NEG	Malonic acid	C00383	1.12	1.29	0.0430
M0103_NEG	Neochlorogenic acid	C17147	3.13	2.34	0.0069
M0112_NEG	Fraxin	-	1.85	3.11	0.0033
M0130_NEG	Scopolin	C01527	2.82	2.06	0.0119
M0173_POS	4-guanidinobutyric acid	C01035	1.51	1.59	0.0012
M0188_NEG	L-tyrosine	C00082	1.18	−0.75	0.0008
M0300_POS	L-isoleucine	C00407	2.83	−0.65	0.0020
M0408_POS	Allopurinol	-	1.64	0.64	0.0340
M0461_POS	N-benzylformamide	C15561	1.27	−0.67	0.0159
M0462_POS	2-hydroxycinnamate	C01772	3.05	−0.64	0.0380
M0506_POS	Isoleucine	-	7.20	−0.62	0.0038
M0866_NEG	2H-1,4-benzoxazin-3(4H)-one	-	1.20	−0.82	0.0216
M0928_POS	2,3-dinor-8-iso prostaglandin F2alpha	C14794	2.76	0.81	0.0237
M1055_POS	Dihydroagathic acid	-	2.05	1.88	0.0024
M1056_POS	Eicosatetraynoic acid	-	3.11	1.92	0.0012
M1096_NEG	Pimelic acid	C02656	1.04	2.38	0.0062
M1221_NEG	N-acetyl-L-phenylalanine	C03519	1.10	−0.89	0.0471
M1261_NEG	Phenylacetaldehyde	C00601	1.24	−0.88	0.0076
M1384_POS	2-pyrazinecarboxamide	-	1.28	2.05	0.0157
M1483_POS	1-naphthalenepentanoic acid	-	1.31	1.53	0.0133
M1527_POS	9-KODE	C14766	5.39	0.93	0.0051
M1715_POS	16-heptadecyne-1,2,4-triol	-	2.51	2.67	0.0323
M1838_POS	Avocadyne 1-acetate	-	6.43	1.46	0.0342
M2118_POS	3-aminosalicylic acid	-	1.42	−1.59	0.0252
M2696_POS	Propentofylline	D01630	1.09	−1.64	0.0125
M2934_POS	3-hydroxycinnamic acid	-	1.00	−0.77	0.0114
M3146_POS	1H-pyrazolo[3,4-b]pyridine-5-carboxamide	-	1.03	−1.80	0.0146

## Data Availability

The transcriptome sequencing data have been deposited to the SRA with the dataset identifier PRJNA911470.

## Supplementary Materials

The following supporting information can be downloaded at: https://www.mdpi.com/article/10.3390/ijms241914840/s1.
