# Supplementary material for: Integrated Analysis of Metabolome and Transcriptome Reveals Insights for Low Phosphorus Tolerance in Wheat Seedling
Source: Int J Mol Sci. 2023 Oct 2;24(19):14840. doi: 10.3390/ijms241914840 (PMC10573437; doi:10.3390/ijms241914840)
Supplement: Supplementary file 1 [file ijms-24-14840-s001.zip › Figure S1.pdf]

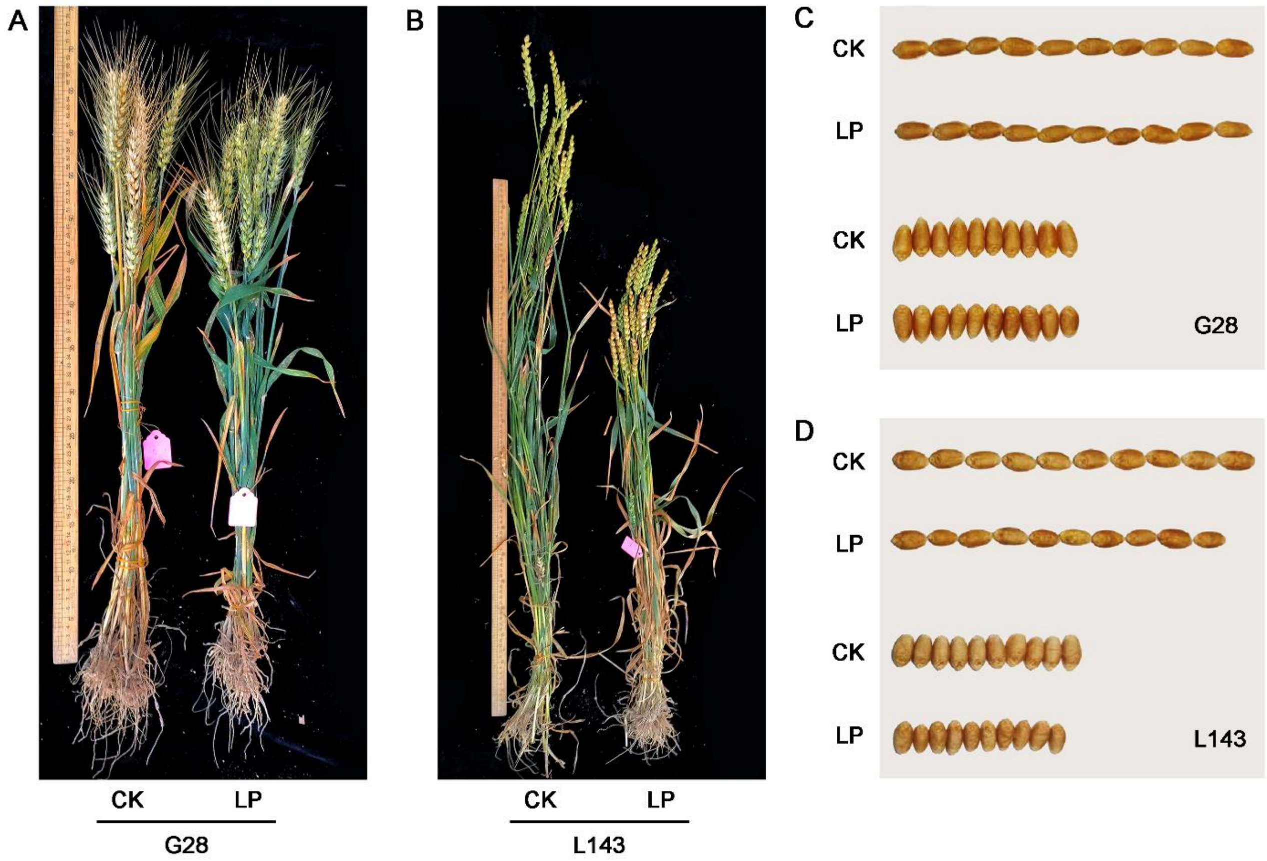

Figure S1. LP-tolerant material G28 and LP-sensitive material L143 under normal and LP conditions behaved at the adult stage. G28 plant height (A) and grain size (C) under LP stress; L143 plant height (B) and grain size (D) under LP stress.
