# Supplementary material for: Integrated Analysis of Metabolome and Transcriptome Reveals Insights for Low Phosphorus Tolerance in Wheat Seedling
Source: Int J Mol Sci. 2023 Oct 2;24(19):14840. doi: 10.3390/ijms241914840 (PMC10573437; doi:10.3390/ijms241914840)
Supplement: Supplementary file 1 [file ijms-24-14840-s001.zip › Figure S3.pdf]

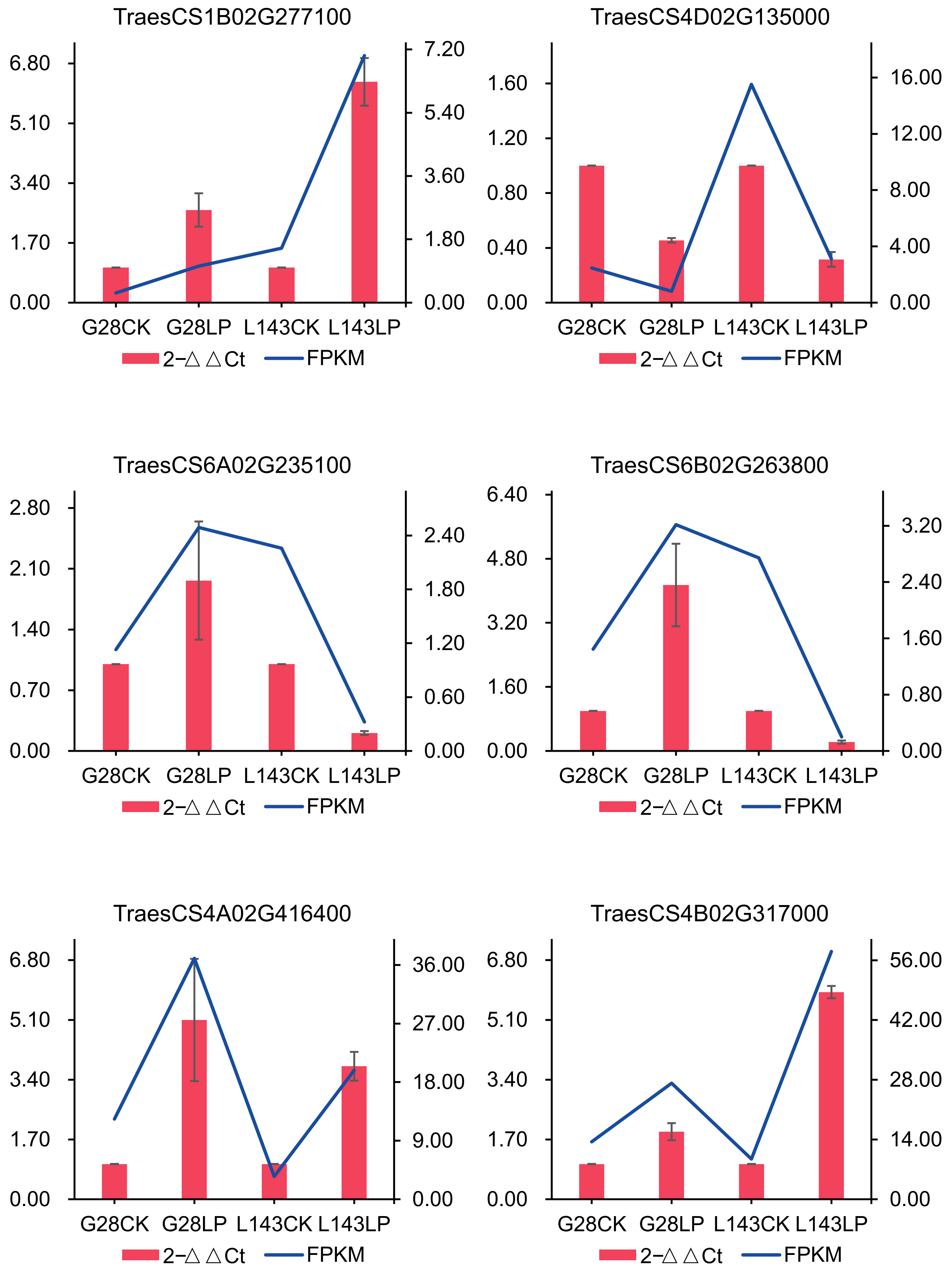
TraesCS1B02G277100
6.80
5.10
3.40
1.70
0.00
7.20
5.40
3.60
1.80
0.00
G28CK G28LP L143CK L143LP
2−△△Ct FPKM
TraesCS4D02G135000
1.60
1.20
0.80
0.40
0.00
16.00
12.00
8.00
4.00
0.00
G28CK G28LP L143CK L143LP
2−△△Ct FPKM
TraesCS6A02G235100
2.80
2.10
1.40
0.70
0.00
2.40
1.80
1.20
0.60
0.00
G28CK G28LP L143CK L143LP
2−△△Ct FPKM
TraesCS6B02G263800
6.40
4.80
3.20
1.60
0.00
3.20
2.40
1.60
0.80
0.00
G28CK G28LP L143CK L143LP
2−△△Ct FPKM
TraesCS4A02G416400
6.80
5.10
3.40
1.70
0.00
36.00
27.00
18.00
9.00
0.00
G28CK G28LP L143CK L143LP
2−△△Ct FPKM
TraesCS4B02G317000
6.80
5.10
3.40
1.70
0.00
56.00
42.00
28.00
14.00
0.00
G28CK G28LP L143CK L143LP
2−△△Ct FPKM

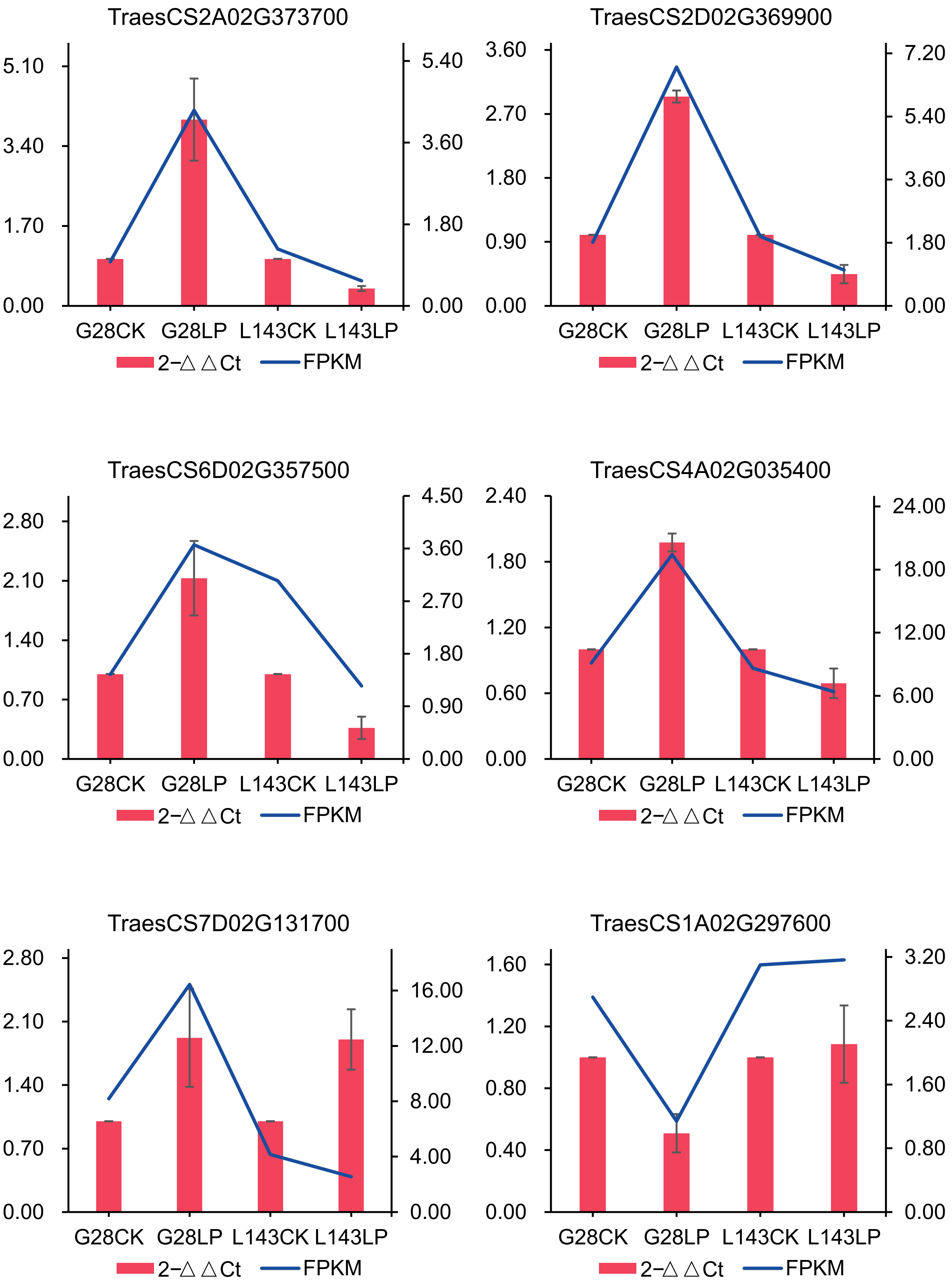

TraesCS2A02G373700
5.10
3.40
1.70
0.00
5.40
3.60
1.80
0.00
G28CK G28LP L143CK L143LP
2−△△Ct FPKM
TraesCS2D02G369900
3.60
2.70
1.80
0.90
0.00
7.20
5.40
3.60
1.80
0.00
G28CK G28LP L143CK L143LP
2−△△Ct FPKM
TraesCS6D02G357500
2.80
2.10
1.40
0.70
0.00
4.50
3.60
2.70
1.80
0.90
0.00
G28CK G28LP L143CK L143LP
2−△△Ct FPKM
TraesCS4A02G035400
2.40
1.80
1.20
0.60
0.00
24.00
18.00
12.00
6.00
0.00
G28CK G28LP L143CK L143LP
2−△△Ct FPKM
TraesCS7D02G131700
2.80
2.10
1.40
0.70
0.00
16.00
12.00
8.00
4.00
0.00
G28CK G28LP L143CK L143LP
2−△△Ct FPKM
TraesCS1A02G297600
1.60
1.20
0.80
0.40
0.00
3.20
2.40
1.60
0.80
0.00
G28CK G28LP L143CK L143LP
2−△△Ct FPKM

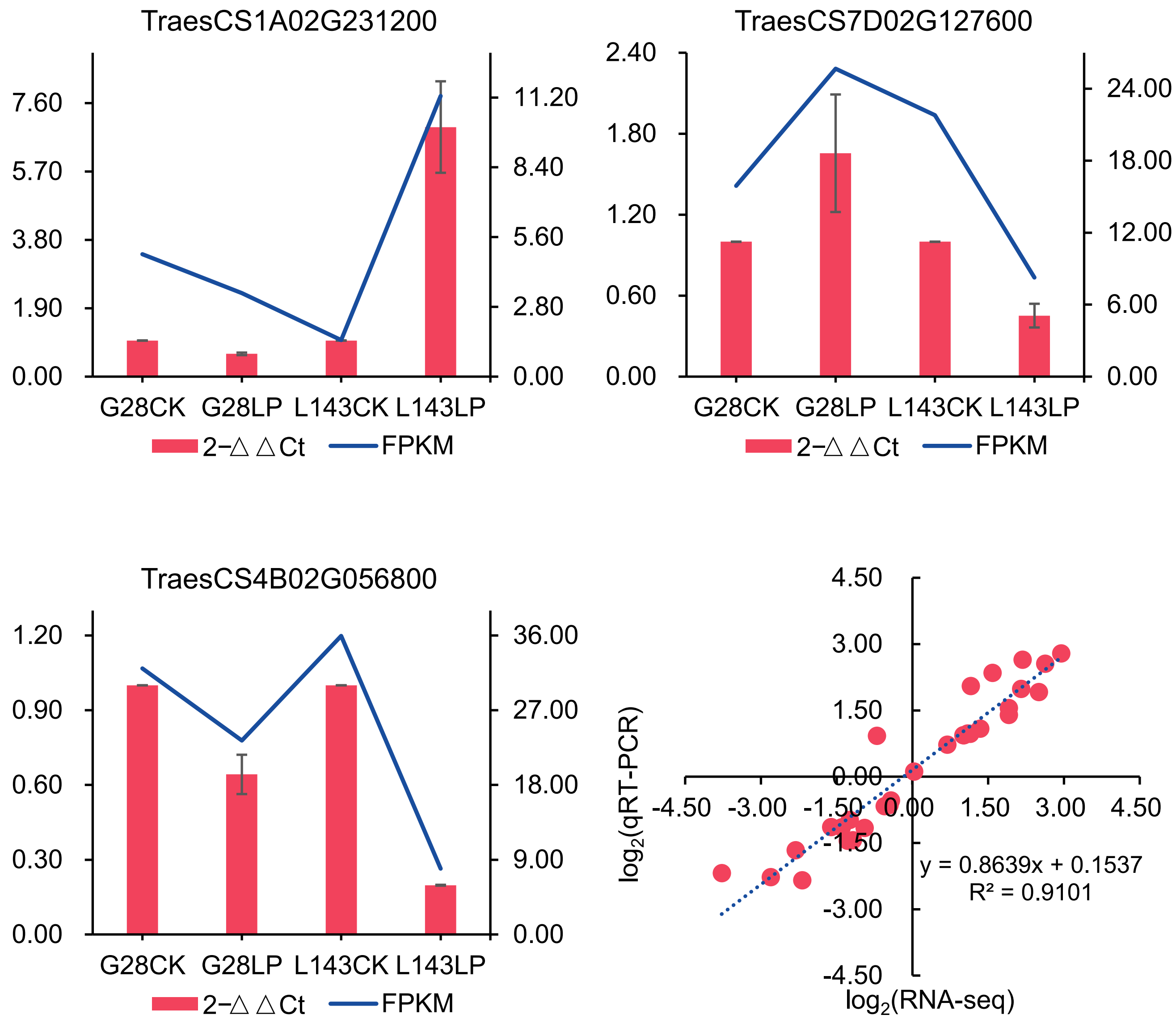


**Figure S3. Comparison of RT-qPCR and RNA-Seq results.** Expression patterns of the 15 genes involved in the LP stress of wheat. Correlation analysis based on RT-qPCR and RNA-Seq data. The RT-qPCR expression levels were calculated as a ratio relative to the level of expression of G28CK and L143CK, which was set as 1. Bars indicate means ± standard error (SE) of at least three independent biological replicates. The line chart shows the gene expression level from the transcriptome (FPKM).
