# Supplementary figures and images for: Integrated Analysis of Metabolome and Transcriptome Reveals Insights for Low Phosphorus Tolerance in Wheat Seedling
Source: Int J Mol Sci. 2023 Oct 2;24(19):14840. doi: 10.3390/ijms241914840 (PMC10573437; doi:10.3390/ijms241914840)

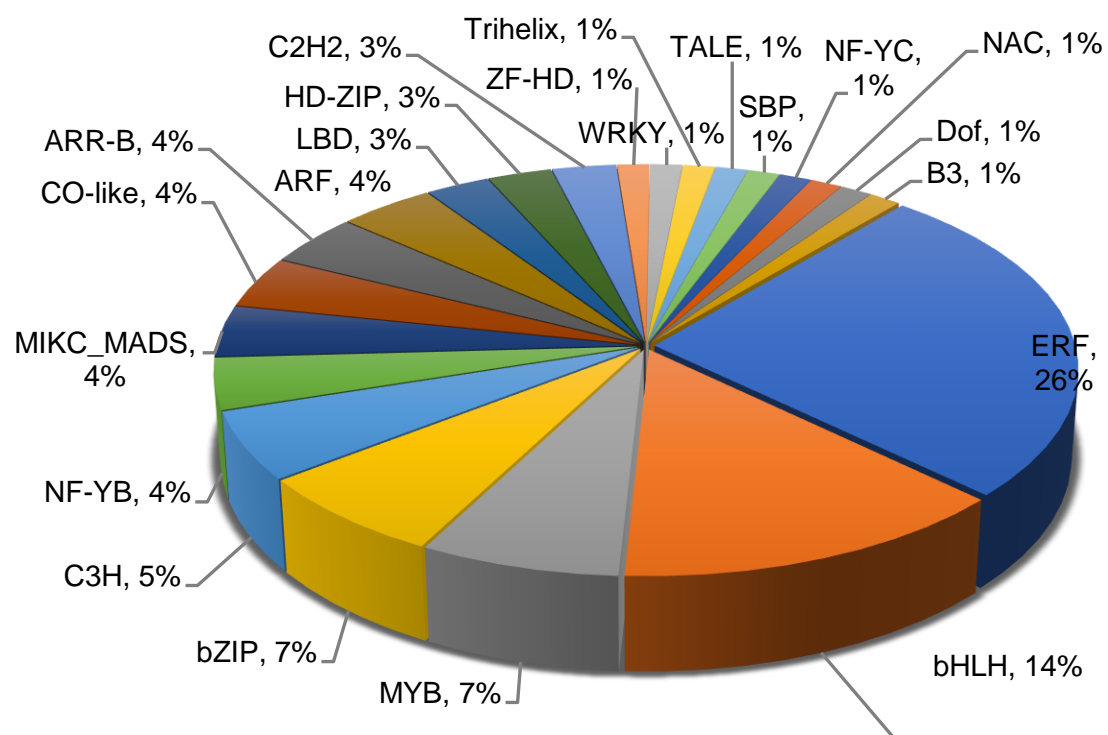

**Figure S2. Class of the 1,120 core DEGs associated with LP stress involved in TFs.**

Supplement: Supplementary file 1 [file ijms-24-14840-s001.zip › Figure S2.pdf]
